# Total bilateral urine collecting system duplication

**DOI:** 10.11604/pamj.2018.29.218.14917

**Published:** 2018-04-23

**Authors:** Souhail Regragui, Amine Slaoui

**Affiliations:** 1 Urology-B-Department, Avicenne Hospital, University Mohammed V, Rabat, Morocco

**Keywords:** Duplex collecting system, kidney, ureter

## Image in medicine

A duplex collecting system, or duplicated collecting system, is one of the most complex congenital urinary tract malformations. It can be a complete split from the pyelocalicea to the ureteral orifice in the bladder, also called total duplication or pyelo ureteral duplicity. Or, the two ureters may unite before emptying into the bladder and define then the partial duplication or bifid ureter. The pathogenesis happens during embryonic life. Indeed, if a single ureteral bud bifurcates before the ampulla's bifurcation, a duplex kidney results with bifid pelvis or bifid ureter. However, if two ureteral buds arise from the Wolffian duct, a duplex kidney results with complete ureteral duplication. Most duplicated collecting systems are asymptomatic and diagnosed incidentally. Symptoms are due to complications such as obstruction, reflux, ureterocoele and infection, and are usually seen in complete duplex urinary tract. Surgical treatment is provided only when symptoms occur. Herein we report a case of bilateral duplex collecting system in a 25 years old woman without significant medical history, presented with a slight lower back pain. CT scan was performed and it set the diagnosis by showing bilateral duplex collecting systems with fully duplicated ureters. It brought out also a moderate hydronephrosis without any obstruction. However, we didn't found any vesicoureteral reflux. The kidney function was normal as well, and the urine culture was negative. The therapeutic strategy was surgical abstention due to the pauci-symptomatic aspect of the lesion. Nonetheless, a closer clinical follow up must be performed.

**Figure 1:**
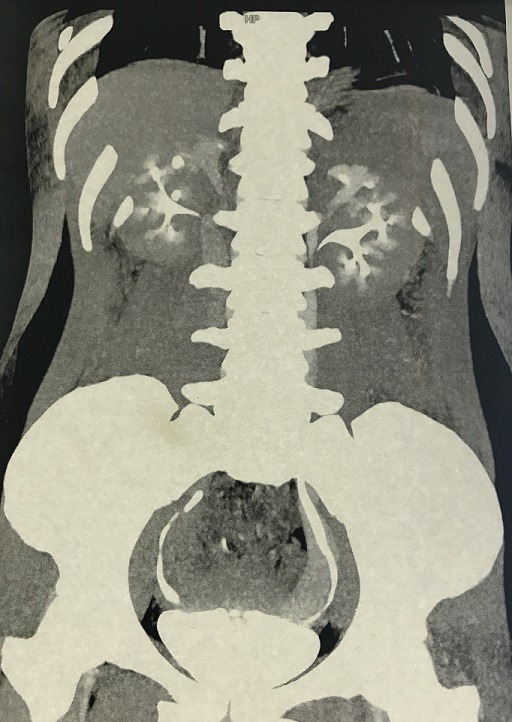
Total bilateral urine collecting system duplication

